# Erectile Dysfunction and Risk of End Stage Renal Disease Requiring Dialysis: A Nationwide Population-Based Study

**DOI:** 10.1371/journal.pone.0102055

**Published:** 2014-07-11

**Authors:** Yuan-Chi Shen, Shih-Feng Weng, Jhi-Joung Wang, Kai-Jen Tien

**Affiliations:** 1 Department of Urology, Kaohsiung Chang Gung Memorial Hospital, Kaohsiung, Taiwan; 2 Cheng Shiu University, Kaohsiung, Taiwan; 3 Department of Medical Research, Chi Mei Medical Center, Tainan, Taiwan; 4 Department of Hospital and Health Care Administration, Chia Nan University of Pharmacy and Science, Tainan, Taiwan; 5 Division of Endocrinology and Metabolism, Department of Internal Medicine, Chi Mei Medical Center, Tainan, Taiwan; 6 The Center of General Education, Chia Nan University of Pharmacy and Science, Tainan, Taiwan; National Health Research Institutes, Taiwan

## Abstract

**Background:**

Previous studies have suggested that erectile dysfunction (ED) is an independent risk factor for macrovascular disease. Very few studies have evaluated the relationship between ED and risk of end stage renal disease (ESRD) requiring dialysis.

**Methods:**

A random sample of 1,000,000 individuals from Taiwan's National Health Insurance database was collected. We selected the control group by matching the subjects and controls by age, diabetes, hypertension, coronary heart disease, hyperlipidemia, area of residence, monthly income and index date. We identified 3985 patients with newly-diagnosed ED between 2000 and 2008 and compared them with a matched cohort of 23910 patients without ED. All patients were tracked from the index date to identify which patients subsequently developed a need for dialysis.

**Results:**

The incidence rates of dialysis in the ED cohort and comparison groups were 10.85 and 9.06 per 10000 person-years, respectively. Stratified by age, the incidence rate ratio for dialysis was greater in ED patients aged <50 years (3.16, 95% CI: 1.62–6.19, p = 0.0008) but not in aged 50–64 (0.94, 95% CI: 0.52–1.69, p = 0.8397) and those aged ≧65 (0.69, 95% CI: 0.32–1.52, p = 0.3594). After adjustment for patient characteristics and medial comorbidities, the adjusted HR for dialysis remained greater in ED patients aged <50 years (adjusted HR: 2.08, 95% CI: 1.05–4.11, p<0.05). The log-rank test revealed that ED patients <50-years-old had significantly higher cumulative incidence rates of dialysis than those without (p = 0.0004).

**Conclusion:**

Patients with ED, especially younger patients, are at an increased risk for ESRD requiring dialysis later in life.

## Introduction

Erectile dysfunction (ED) is defined as the inability to achieve or maintain an erection sufficient for sexual performance [1]. ED is an important worldwide issue, affecting 152 million men in 1995 and 322 million men in 2025 and negatively impacts life quality, self-esteem, and intimacy [2], [3]. One telephone survey of ED prevalence in Taiwan reported that nearly thirty percent of men over 30 years had ED [4]. This medical problem has been associated with endothelial dysfunction and low-grade vascular inflammation and often clusters with hypertension, diabetes mellitus, hyperlipidemia, obesity, and metabolic syndrome [5], [6], [7]. In addition, it has been reported ED as an independent risk factor for macrovascular diseases such as coronary heart disease, cerebrovascular disease (CVD), and peripheral artery disease [8], [9], [10], [11], [12], [13].

ED has been extensively studied in relation to metabolic disease and CVD but rarely in relation to microvascular diseases such as end stage renal disease (ESRD). Although ED is commonly found in patients in a late stage of chronic kidney disease (CKD) [14], little attention has been paid to whether it is related to the progression of kidney disease. Several studies have found that there is strong association between CVD and ESRD and that the interaction between the two diseases is bidirectional and complex [5], [15], [16], [17]. Because CVD and ESRD may share a common pathophysiological pathway, it is possible that ED might also be associated with the development of ESRD. One cross-sectional study by Hermans et al., investigating the relationship between ED and microangipathy in diabetes, found ED to be associated with retinopathy but not with level of estimated glomerular filtration rate (eGFR) [18], though another cross-sectional investigation by Chuang et al. reported it to be associated with both albuminuria and eGFR [19]. In a meta-analysis by Navaneethan et al., the prevalence of ED in chronic kidney disease was 70%, though one of the limitations with that review was that the studies reviewed were of small sample sizes and had cross-sectional designs [20].

In this longitudinal follow-up study, we used a population-based national insurance dataset in Taiwan to examine the relationship between ED and the risk of ESRD requiring dialysis.

## Methods

### Data sources

Taiwan launched a single-payer National Health Insurance (NHI) program on March 1, 1995. The NHI database covers nearly all of Taiwan's population, and is one of the largest and most complete population-based datasets in the world. The data used in this study came from the Longitudinal Health Insurance Database 2000 (LHID2000), which is a sub-set of NHI database that contains all claims data (from 1996 to 2011) of one million beneficiaries. This sample was systemic-randomly selected in 2000. There are no significant differences in age, gender and health care costs between the sample group and all enrollees in the NHI program. The LHID2000 provides encrypted patient identification numbers, gender, date of birth, dates of admission and discharge, the ICD-9-CM (International Classification of Diseases, Ninth Revision, Clinical Modification) codes of diagnoses and procedures, details of prescriptions, registry of Catastrophic Illness Patient Database, as well as costs covered and paid for by NHI. The institutional review board of Chi Mei Medical Center approved the protocol of this study. Informed consent was not required because the datasets were devoid of identifiable personal information.

### Study sample

A retrospective cohort study was conducted with two study groups: a newly onset ED (erectile dysfunction) group and a matched non-ED control group during the recruitment period of 2000–2008. ED was defined in a patient if he had (1) at least two outpatient service claims with the codes of ED (ICD-9-CM code 60784) at any hospital or local medical clinic or (2) any one single hospitalization with ED listed among the five claims diagnosis codes. Patients diagnosed as having ED before 2000 were excluded. CKD is a well-known strong predictor for ESRD requiring dialysis. To evaluate the true association between ED and ESRD requiring dialysis, any patient with a diagnosis of renal function impairment (ICD9CM code: 582, 583, 585, 586, 588) was excluded. Because the outcome of interest was the occurrence of ESRD requiring dialysis, we also excluded patients identified in the registry of Catastrophic Illness Patient Database as being diagnosed as having chronic kidney disease (ICD-9-CM code 585), which would indicate that they were receiving dialysis for ESRD before their diagnosis of ED.

For each ED patient, six patients not diagnosed with ED were randomly selected from the dataset as a control group match. ED patients were matched with members of the control group by age, diabetes, hypertension, coronary heart disease, hyperlipidemia, area of residence, monthly income and index date. The index date for the ED subjects was the date of their first registration. The year of that index date was used to create the index date for each comparison subject. Demographic data such as gender, age, geographic area of Taiwan, and monthly income (record as NT$) were collected. Baseline co-morbidities of these patients were also recorded. These included diabetes mellitus (ICD9CM code:250), hypertension (ICD9CM code:401–405), coronary heart disease (ICD9CM code:410–414), and hyperlipidemia (ICD9CM code:272), because these co-morbidities are known to affect the risk of ESRD. We counted any of these comorbid conditions if the condition was diagnosed in an inpatient setting or in three or more ambulatory care claims coded one year before the index medical care date. Follow-up time in person-years (PY) was calculated for each person until diagnosis of ESRD requiring dialysis, death, or the end of 2011.

## Statistical Analyses

All statistical operations were performed using the SAS 9.3.1 statistical package (SAS Institute, Inc., Cary, North Carolina, USA). Pearson's χ2 tests was used to compare differences in the baseline characteristics, co-morbid medical disorders and socio-demographic status between the study and control cohort. The incidence rate was calculated as the number of ESRD requiring dialysis cases during the follow-up, divided by the total person-years for each group by age and duration. The risk of being diagnosed as having ESRD requiring dialysis was compared between the ED group and the control group by estimating the incidence rate ratio using Poisson regression. Moreover, stratified Cox proportional hazard regression (stratified by age group <50 and ≧50) analysis was used to compute the adjusted hazard ratio for developing ESRD requiring dialysis between patients with and without ED after adjusting for possible confounding factors (diabetes, hypertension, coronary heart disease, hyperlipidemia, geographic area and monthly income). Kaplan-Meier analysis was also used to calculate the cumulative incidence rates of ESRD requiring dialysis between two cohorts, and the log-rank test was used to analyze the differences between the survival curves. A two sided P value <0.05 was considered significant.

## Results

As can been seen in Table 1, a summary of the baseline characteristics and comorbid conditions of the two groups, 3985 male ED patients and 23910 non-ED patients were enrolled in the study. Because we selected the control group by matching with patients' characteristics and comorbidities, there were no significant differences in diabetes, hypertension, coronary heart disease, hyperlipidemia, geographic area and income.

**Table 1:** Demographic characteristics and comorbid medical disorders for patients with and without erectile dysfunction (ED) in Taiwan.

Category	Subcategory	Patients with ED (N = 3985)	Patients without ED (N = 23910)	P-value
Age	0–49	1654(41.51%)	10063(42.09%)	0.7350
	50–64	1475(37.01%)	8818(36.88%)	
	≧65	856(21.48%)	5029(21.03%)	
Diabetes	Yes	541(13.58%)	3058(12.79%)	0.1704
	No	3444(86.42%)	20852(87.21%)	
Hypertension	Yes	905(22.71%)	5422(22.68%)	0.9628
	No	3080(77.29%)	18488(77.32%)	
Coronary heart disease	Yes	327(8.21%)	1935(8.09%)	0.8090
	No	3658(91.79%)	21975(91.91%)	
Hyperlipidemia	Yes	370(9.28%)	2079(8.70%)	0.2233
	No	3615(90.72%)	21831(91.30%)	
Area	North	2143(53.78%)	13387(55.99%)	0.0640
	Central	740(18.57%)	4267(17.85%)	
	South	1003(25.17%)	5729(23.96%)	
	East	99(2.48%)	527(2.20%)	
Income	NT<15840	1801(45.19%)	10646(44.53%)	0.4754
	NT 15841∼25000	988(24.79%)	5858(24.50%)	
	NT>25001	1196(30.01%)	7406(30.97%)	

Table 2 shows the risk for ESRD requiring dialysis in all patients stratified by age and number at risk of follow-up years. During the follow-up period, the incidence rates for ESRD requiring dialysis for the ED and non-ED cohorts were 10.85 and 9.06 per 10000 person-years, respectively. Stratified by age, the younger group <50 years has the most pronounced (IRR  = 3.16, 95% CI  = 1.62–6.19, p = 0.0008). We found no group differences in risk for ESRD requiring dialysis in the age groups 50–64 years and ≥65 years (IRR  = 0.94, 95% CI  = 0.52–1.69, p = 0.8397 and IRR  = 0.69, 95% CI  = 0.32–1.52, p = 0.3594, respectively). Analyzing by number at risk of follow up years, we found that the IRR for ESRD requiring dialysis increased progressively but not significantly along with the duration of ED.

**Table 2:** Risk for end stage renal disease (ESRD) requiring dialysis in patients with and without erectile dysfunction (ED).

		Patients with ED				Patients without ED				IRR (95% CI)	P value
		N	ESRD requiring dialysis	PY#	Rate*	N	ESRD requiring dialysis	PY#	Rate*		
All		3985	33	30412.37	10.85	23910	162	178837.16	9.06	1.20(0.82–1.74)	0.3445
Age	<50	1654	13	12530.70	10.37	10063	25	76264.65	3.28	3.16(1.62–6.19)	0.0008
	50–64	1475	13	11206.17	11.60	8818	80	64914.22	12.32	0.94(0.52–1.69)	0.8397
	≧65	856	7	6675.50	10.49	5029	57	37658.29	15.14	0.69(0.32–1.52)	0.3594
Follow up years	0–2	3985	1	7942.73	1.26	23910	14	47482.70	2.95	0.43(0.06–3.25)	0.4110
	2–4	3955	7	7694.23	9.10	23566	39	45660.37	8.54	1.07(0.48–2.38)	0.8778
	≧4	3575	25	14775.41	16.92	21114	109	85694.09	12.72	1.33(0.86–2.05)	0.1982

#PY, person-years.

*Rate: per 10000 person-years.

We further analyzed the crude and adjusted HR for ESRD requiring dialysis in patients aged <50 years and those aged ≧50 (Table 3). After adjusting for age, gender, DM, HTN, CHD, hyperlipidemia, geographic area and monthly income, we found ED patients aged <50 years to be 2.08 times more likely to develop ESRD requiring dialysis (adjusted HR  = 2.08, 95% CI  = 1.05–4.11, P<0.05). There were no group differences in crude and adjusted HRs for ESRD requiring dialysis in those aged ≧ 50 years.

**Table 3:** Crude and adjusted hazard ratios (HR) for the development of end stage renal disease (ESRD) requiring dialysis among the sample patients stratified by age.

		Age <50		Age≧50	
		Crude HR (95% CI)	Adjusted HR (95% CI)	Crude HR (95% CI)	Adjusted HR (95% CI)
Erectile dysfunction	Yes	3.17*(1.62–6.19)	2.08*(1.05–4.11)	0.83(0.52–1.33)	0.85(0.53–1.37)
	No	1.00	1.00	1.00	1.00

*P-value <0.05.

Adjusted for diabetes, hypertension, coronary heart disease, hyperlipidemia, geographic area and monthly income.

**Figure 1:**
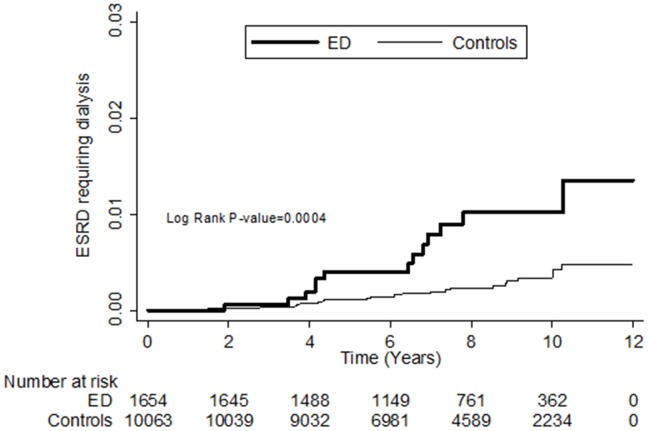
The cumulative incidence rate for end stage renal disease (ESRD) requiring dialysis for patients <50 years of age with erectile dysfunction (ED) and without ED (log-rank P value  = 0.0004).

The results of Kaplan-Meier analysis revealed that ED patients <50 years old had a higher cumulative incidence rate for ESRD requiring dialysis than the comparison cohort (log-rank test P = 0.0004) (Figure 1).

## Discussion

Many studies have reported ED to be associated with macrovascluar disease, but few have investigated the impact of ED on the progression of a microvascular disease such as kidney disease. This study represents the first nationwide population-based study to investigate the relationship between ED and risk for ESRD requiring dialysis in an Asian population. We found the incidence rate for ESRD requiring dialysis to be 10.85 per 10000 person-years in patients with ED. The risk was especially greater in younger ED patients of age <50 years. After adjusting for patient characteristics and medical comorbidities, we did not find an association between ED and increased risk in patients of age ≧50. The crude and adjusted HR for ESRD dialysis was 3.17-times and 2.08-times higher risk in ED patients of age <50 years than it was in the comparison cohort, respectively. Although the HR decreased after adjustment, ED patients remained at significantly higher risk.

Several studies have investigated the association between ED and microvascular disorders. One cross-sectional study by Siu et al., investigating the prevalence and risk factors of ED in diabetic patients [21], found a significant association between ED and microangiopathy and reported the odd ratios for retinopathy, microalbuminuria, clinical proteinuria and sensory neuropathy to be 2.27, 1.68, 2.27, and 2.05, respectively. Another small cross-sectional study involving 82 male diabetics in Japan reported an association between ED and proteinuria [22], though that study did not address the relationship between ED and eGFR. One study of 221 male diabetes reported an association between ED prevalence of elevated albuminuria [18], but found no difference in eGFR level between patients with ED and those without. Chuang et al., conducting a cross-sectional study to evaluate the association between ED and severity of albuminuria [19], reported that not only was ED associated presence of albuminuria and low eGFR level, but it had a stronger association on the presence of macroalbuminuria than microalbuminuria (odd ratios 4.49 and 2.28, respectively). ED is a common feature of CKD and ESRD. Two studies have closely correlated ED to the stage of CKD [14], [20]. Although their results differed somewhat, both studies reported an association between ED and microangiopathy and suggested that it might have an impact on the disease progression. ED is generally thought to be secondarily increased in subjects with CKD and ESRD. However, a cross-sectional study design cannot be used to infer directionality. One important step to clarify the direction of the putative ED-ESRD association is the performance of a longitudinal study of individuals initially free of ESRD at baseline. Because CKD is an extremely strong predictor for ESRD requiring dialysis, we also excluded patients diagnosed as having any form kidney disease. Our study used ESRD requiring regular dialysis as the end point to evaluate the influence of ED on the progression of kidney disease.

Although the underlying mechanism contributing to the association between ED and risk of ESRD requiring dialysis is likely complex, there are some possible explanations that can be considered. First, metabolic comorbidities and cardiovascular disease are highly prevalent in ED patients. One national database in United States reported the crude prevalence rates to be 41.6% for hypertension, 42.4% for hyperlipidemia and 20.2% for diabetes in ED patients [5]. These metabolic comorbidities are the traditional risk factors that can deteriorate renal function. Since metabolic comorbidities were matched and adjusted for in the present study, the relationship between ED and ESRD requiring dialysis survived. The association between ED and ESRD may be in part due to these metabolic comorbidities, but they cannot explain the entire relationship between ED and ESRD. Second, ED is considered as a surrogate marker of endothelial damage, which is an important pathologic change in ED [23]. The hallmark of endothelial dysfunction is reduced synthesis of nitric oxide (NO), increased vasoconstrictor peptide endothelin-1 (ET-1), and increased production of the asymmetric dimethylarginine (ADMA) and inflammatory cytokines, including interleukin-6 (IL-6), C-reactive protein (CRP) and tumor necrosis factor-a (TNF-a) [24]. NO reduction was found to result in increased glomerular capillary pressure, vascular resistance and decreased capillary ultrafiltration in one animal study [25], suggesting that long term endothelial dysfunction might result in irreversible kidney damage. One study by Ravani and colleagues suggested that ADMA is inversely related to GFR and represents a strong and independent marker for progression to ESRD [26]. A population-based cohort study of up to 4926 patients with 15 years of follow-up reported that elevations of most inflammation markers, including TNF-a receptor 2 and IL-6 level, could predict risk of CKD [27]. Therefore, ED might be considered as an early marker and factor predisposing an individual to ESRD and not just diseases secondary to ESRD only.

In the current study, individuals who were diagnosed with ED later in life were at much less risk for ESRD requiring dialysis. The reason for this difference is not known. We speculate this lack of association in an elderly population may be due to several reasons. Compared with ED, the traditional risk factors for ESRD, which include hypertension, hyperlipidemia and diabetes, may have more impact on the disease progression. Marumo et al. evaluated the age-related prevalence of ED and the comorbidities to be common in ED patients in a cross-sectional study [28], and found the prevalence of hypertension, hyperlipidemia and diabetes to be dramatically increased after the age of 50 years. In the present study, the lack of association between ED and ESRD in elderly population may be due to by our adjustment for confounders in our model or may be due to the fact that elderly people with ESRD in Taiwan often refuse chronic dialysis therapy. In addition, the incidence of ESRD requiring dialysis may be underestimated in the elderly population.

This study has some limitations. First, the diagnosis of ED was based on diagnostic codes recorded by the physician in the NHI claims forms; therefore, some registration bias might be involved. The identification of ED and ESRD requiring dialysis diagnoses were based on diagnoses listed in an administrative database and therefore may be less accurate than diagnoses undertaken individually following standard procedures. In Taiwan, the self-administered International Index of Erectile Dysfunction (IIEF-5) questionnaire is usually used to assess and quantify the patients' subjective symptoms of ED. The diagnosis as well as the severity of ED can be evaluated based on the results of self-administered IIEF-5 questionnaire. Because the patients' raw questionnaire scores were not available in the administrative database, we defined ED by ICD-9-CM codes. To ensure the accuracy of these diagnostic codes, only the ED patients diagnosed by two patient claims records citing ED a primary diagnosis were recorded. ESRD requiring dialysis is definitely registered in Catastrophic Illness Patient Database in Taiwan. Patients identified in the registry of Catastrophic Illness Patient Database as having diagnosis of chronic kidney disease (ICD-9-CM code 585), which would indicate that they were receiving dialysis for ESRD. Therefore, registration bias was minimized as much as possible in this study. Another limitation may be related to lack of willingness to discuss possible ED problems in Taiwan. It is possible that some of the controls may have ED but not yet diagnosed. If this were the case, our results would have been biased toward the null leading to a more conservative estimate of association. Another limitation is that disease severity could not be assessed from our dataset. Still another limitation was claims records do not include smoking, Chinese herbal medicine, occupation, and family histories. Nor are environmental data recorded. Despite these limitations, the study is strong because it has a longitudinal, large population-based design and covers nearly all of Taiwan's residents. As such, both referral and selection biases are minimized.

In conclusion, patients with ED, which is conventionally considered as a risk factor of cardiovascular disease, are at increased risk for ESRD, especially in younger adults. Patients with early diagnosed ED are at an increased risk for ESRD with dialysis later in life. Further studies are needed to evaluate the possible underlying mechanisms between these two conditions.
